# Role of the internet of medical things in care for patients with interstitial lung disease

**DOI:** 10.1097/MCP.0000000000000971

**Published:** 2023-05-19

**Authors:** Gizal Nakshbandi, Catharina C. Moor, Marlies S. Wijsenbeek

**Affiliations:** Department of Respiratory Medicine, Erasmus University Medical Centre, Rotterdam, The Netherlands

**Keywords:** eHealth, interstitial lung disease, internet of things

## Abstract

Online technologies play an increasing role in facilitating care for patients with interstitial lung disease (ILD). In this review, we will give an overview of different applications of the internet of medical things (IoMT) for patients with ILD.

**Recent findings:**

Various applications of the IoMT, including teleconsultations, virtual MDTs, digital information, and online peer support, are now used in daily care of patients with ILD. Several studies showed that other IoMT applications, such as online home monitoring and telerehabilitation, seem feasible and reliable, but widespread implementation in clinical practice is lacking. The use of artificial intelligence algorithms and online data clouds in ILD is still in its infancy, but has the potential to improve remote, outpatient clinic, and in-hospital care processes. Further studies in large real-world cohorts to confirm and clinically validate results from previous studies are needed.

**Summary:**

We believe that in the near future innovative technologies, facilitated by the IoMT, will further enhance individually targeted treatment for patients with ILD by interlinking and combining data from various sources.

## INTRODUCTION

Interstitial lung diseases (ILDs) are a heterogeneous group of rare chronic lung diseases, characterized by inflammation and/or fibrosis. ILD can broadly be classified as exposure related, including drug-use, auto-immune related, idiopathic, sarcoidosis, and ultra-rare diseases [1,2]. Some of these diseases are reversible, some have a stable disease course, but many forms of ILD are often progressive and associated with high morbidity and mortality. Symptoms as dyspnea, impaired exercise tolerance, cough and fatigue often lead to an impaired quality of life. Disease course of ILD in individual patients is currently difficult to predict, which makes close monitoring essential. Frequent outpatient clinic visits are needed to follow up on lung function and symptoms, initiate medication, and assess the effects and adverse effects of treatment [3]. These visits can be burdensome for patients with increasing symptoms. Moreover, travel distances are often long, as in many countries care for patients with these rare diseases is organized in expert centers. 

**Box 1:**
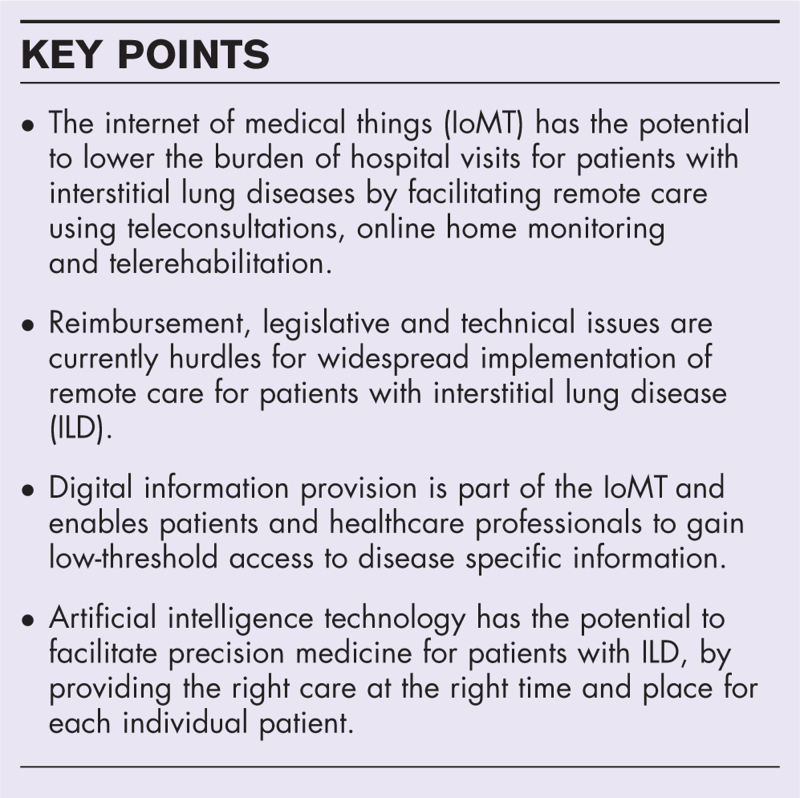
no caption available

Hence, there has been increasing interesting in innovative (online) technologies to improve care for patients with ILD [4,5]. The internet of medical things (IoMT) is an umbrella term for the multiple layered technological infrastructure that enables the connection of software applications and medical devices with technological healthcare systems [6]. The IoMT enables healthcare professionals to monitor patients from a distance, but also provides patients possibilities for self-monitoring and contributes to inter-colleague communication.

**FIGURE 1:**
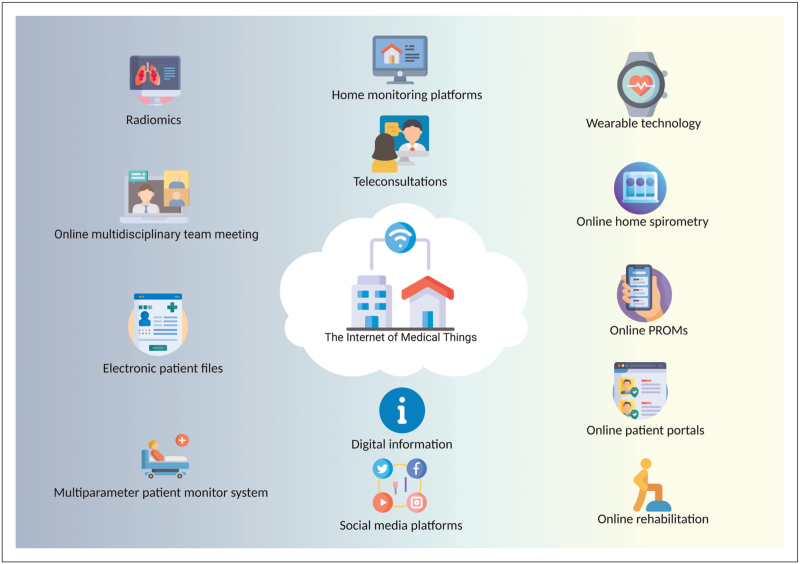
Overview of the different applications of the internet of medical things for patients with interstitial lung diseases. Remote applications: wearable technology, online home spirometry, online patient reported outcome measures, online patient portals, online rehabilitation. Outpatient clinic applications: home monitoring platforms, teleconsultations, digital information, social media platforms. In-hospital applications: radiomics, online multidisciplinary team meetings, electronic patient files, multiparameter patient monitor systems.

Examples of IoMT for patients with ILD include teleconsultations, home monitoring of physical parameters, symptoms and quality of life, online pulmonary rehabilitation, and self-management programs, digital information provision, but also in-hospital applications such as artificial intelligence (AI) algorithms and online multidisciplinary team meetings. In this review, we will provide an overview of the different applications of the IoMT for patients with ILD (Fig. 1).

## TELECONSULTATIONS

Teleconsultation refers to the delivery of healthcare services using information and communication technologies at a distance, such as electronic, telephone and video consultations [7,8]. Compared to consultations by telephone, an advantage of video consultation is the ability to see patients and their environment. This can help better assess their physical condition and it enables a more natural face-to-face interaction [8]. Many healthcare professionals specialized in ILD are positive towards the implementation of video consultations for follow up of patients, and believe video consultations can facilitate better access to care, especially for patients that live further away from ILD specialized centers [8]. During the coronavirus disease 2019 (COVID-19) pandemic, outpatient clinic visits have been replaced by video consultations in some centers worldwide [4,8,9^▪^]. Importantly, video consultations may not be a suitable option for all patients and in all phases of the disease, such as the diagnostic phase, during acute worsening of disease, or following medication changes [8,10,11]. Although the COVID-19 pandemic has boosted the use of video consultations for patients with ILD, broad implementation in daily clinical practice still needs optimization. Recently, a comment from the United States of America (USA) on the application of teleconsultations demonstrated the current legislative barriers. In some parts of the USA, patients have to be in the same state as where their treating healthcare professional holds a license. This hampers the use of teleconsultations across states and may result in situations where patients have to travel for several hours to have a video consultation with their ILD specialist [9^▪^]. The fact that these laws vary from state to state creates a barrier to achieving uniform and widespread implementation. Besides these legislative issues, technical aspects also play a role. Not all patients are able to conduct teleconsultation due to lack of internet access or compatible devices. Also, teleconsultation can impact the interaction between healthcare professional and patient. Some nuances of patient interaction, or unexpected issues may be attenuated during teleconsultations. Another downside of teleconsultation may be that healthcare professionals lack access to physical parameters, such as lung function and pulse oximetry.

## ONLINE HOME MONITORING

Online home monitoring of physical parameters broadens the possibilities of remote care for patients with ILD, and can complement video consultations. The IoMT provides the opportunity to collect and monitor health data of patients with smart devices that are linked to secured online applications. Online home monitoring has the potential to be used throughout the disease course of patients with ILD, for example for close monitoring of disease behavior, guidance of pharmacological and nonpharmacologic treatment decisions, and early detection of disease progression [12^▪▪^].

### Home spirometry

**FIGURE 2:**
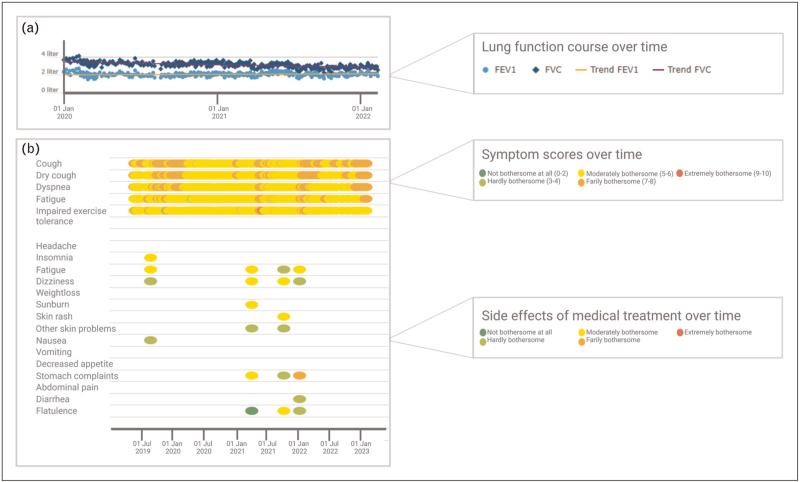
Example of an online home monitoring program for patients with interstitial lung diseases. (a) Online overview of lung function course over time. (b) Online overview of patient reported outcome measurements over time.

Home spirometry, using a hand-held spirometer, is the most frequently studied home monitoring application for patients with ILD, as forced vital capacity (FVC) is one of the main outcomes used to monitor disease course [13–19]. The first studies, in patients with IPF, used paper diaries to collect home spirometry results, which hampers use in clinical practice [18,20]. Moreover, some studies encountered technical problems, such as connection issues with the spirometers, and large variability of the data [21^▪▪^,^22^]. Many of these problems can be overcome by using the IoMT. The use of spirometers linked to an online application facilitates real-time access to lung function results and quality checks of the measurements (Fig. 2a). Studies have confirmed reliability and accuracy of online home spirometry, by comparing results with in-hospital values [13,23,24]. Ongoing trials investigate the role of home spirometry for early detection of acute exacerbations and prediction of disease behavior, and evaluation of response to treatment in different forms of ILD [25–27].

Since the COVID-19 pandemic, home spirometry has been increasingly used in daily clinical practice to ensure continuity of care [4,28]. This eventually leads to very large amounts of FVC data being collected, which makes it impossible to manually check all data real-time and provide immediate feedback to the patient. AI has been proposed as one of the solutions to help identify poor quality lung function measures, assist with the interpretation of the home spirometry results, and send automatically generated alerts to healthcare providers in case of relevant FVC decline, though this needs further study in the home setting [29^▪▪^,^30^].

### Wearables

Hypoxemia is one of the main problems of patients with ILD, and associated with poor prognosis [31,32]. In current practice, the in-hospital 6-min walking test (6MWT) is a tool to assess functional exercise capacity of patients and hypoxemia on exertion. Based on the results, supplemental oxygen therapy may be indicated. Even though the 6MWT provides valuable information on the general health status of the patient, the functional exercise capacity measured with the 6MWT may not be representative for regular daily activity of patients at home. A possibly more representative way to monitor physical activity and hypoxemia during daily activity is by using wearable technology [33].

Wearables are any sort of electronic device that can be worn by the user, and collects data on physiological parameters such as activity, oxygen saturation, or environmental exposure using in-built sensors [34]. A pilot study in ILD showed that it is feasible to use wearable technology for monitoring oxygen saturation and activity at home. In patients not using long-term oxygen therapy, a high correlation was found between the percentage of time spent with an SPO2 below 90% measured with a portable oximeter at home versus in-hospital 6MWT [33]. Moreover, steps per day measured with a pedometer highly correlated with 6MWT. Another pilot study in patients with sarcoidosis showed that wearing an activity tracker was associated with improvement in exercise capacity and reduced fatigue, even without a personal coaching program [35]. Patients had access to their own results which may have stimulated patients to be more active. A study in IPF found a high correlation between daily physical activity and 6MWT, but they also encountered technical and analytical hurdles, such as data loss and high variability in steps per day [36]. The clinical significance of change in physical activity at home, long-term use and adherence has not yet been studied in ILD. Therefore wearable technology assessing activity has currently no role in clinical practice.

Besides patient specific characteristics, wearable technology can be used to collect real-time environmental data. Air pollution is thought to be associated with the development of ILD, but also with disease progression and higher mortality [37,38]. Two studies found a significant correlation between air pollution and acute exacerbation, and lower lung function in patients with IPF [39,40]. These studies were conducted by prospectively measuring air pollution in the residential area by using the geocode of the patient. However, in these studies patients were not tracked real-time. A study in sarcoidosis analyzed local weather, and found a specific seasonal distribution of peak incidence of sarcoidosis in the United States and Europe [41]. Wearable technology may provide the opportunity to more accurately assess impacts of environmental and seasonal effects on ILD.

### Patient reported outcome measures

Patient reported outcome measurements (PROMs) are frequently used in the ILD research field to assess and monitor the impact of the disease on patients’ symptoms, quality of life, and functional capacity. The use of PROMs can also hold benefits in daily clinical practice. PROMs can assist with the shift towards more value-based healthcare by efficiently identifying problem areas, which may otherwise be neglected. This could help to better structure regular outpatient clinic visits [42]. In some hospitals, PROMs are included in the electronic patient record, but PROMs can also be integrated in more comprehensive online home monitoring programs alongside collection of physical parameters. Evidence shows that it is not needed to validate existing questionnaires if only minor modifications are made to the original measure, as the psychometric properties of the original PROM will still hold for the digital version [43]. Another benefit of digital PROMS are that results can be visualized over time, which helps interpretation and can stimulate patients’ self-management (Fig. 1b). Importantly, some PROMs are lengthy and may be difficult for patients to complete. Visual analogue scales (VAS) could be a good replacement for longer questionnaires. These short one-item questionnaires have shown good correlation with longer quality of life questionnaires in IPF, and can easily be completed online [44,45].

## TELEREHABILITATION

In ILD, pulmonary rehabilitation is associated with improvement of exercise capacity, dyspnea and HRQoL [46]. Telerehabilitation is emerging as an alternative to improve access and adherence to pulmonary rehabilitation programs and has been investigated in several pilot studies in ILD [47]. The potential of telerehabilitation for different chronic lung disease is discussed elsewhere in this issue (Khor and Cox).

## DIGITAL INFORMATION

An important function of the IoMT is providing medical information. Hospitals, patient associations, social media groups, and support groups, share disease-specific information and updates on developments in the field of ILD, using different online platforms. Digital information may help gain better insights in disease course, and enhance self-management. Nevertheless, finding reliable online resources can be difficult for patients, due to the extensive quantity and sometimes incorrect information available [48]. Unfortunately, there are currently no methods available for patients to identify online resources that provide good quality information [48]. Recently, two studies evaluated the quality of information available in YouTube videos and found that in the majority of the videos, the quality was not up to standards [49,50^▪^].

Another, more interactive, way of digital information dissemination are social media channels. Social media platforms can be used for peer support, information exchange, and raising awareness. Different patient organizations are active on social media platforms such as Twitter, Facebook, Instagram, and LinkedIn. These accounts are mainly focused on advocacy, education programs, peer support, and research funding [51,52]. Besides the traditional advantages of connecting patients, social media has also become base for healthcare professionals and researchers to share the latest developments in the ILD research field. However, social media use also has several downsides. Lack of tech savviness, misinformation, and patient privacy are mentioned as potential challenges of use of social media [52].

When it comes to processing digital information, not only patients, but also healthcare professionals struggle with the exorbitant amount of information available. Of note, the medical knowledge doubling time decreased from approximately 50 years in 1950 to several months nowadays [53]. This makes it no surprise that guidance on accurate disease-specific online resources has been identified as important need by ILD healthcare professionals [54]. Currently, different websites are available where ILD healthcare providers can get easy and free access to valuable information, such as pneumotox.com(drug-induced lung diseases) and HPlung.com(listing known exposures associated with hypersensitivity pneumonitis) [55,56]. The maintenance of these websites, which includes technical updates, quality checks and continuous provision of new information is an effortful task. Novel AI tools could possibly help be a solution for automatically identifying and updating high quality information.

## IN-HOSPITAL APPLICATIONS OF INTERNET OF MEDICAL THINGS

The IoMT is not limited to remote care, but also concerns in-hospital processes, for example electronic patient records, continuous patient monitoring systems used in intensive care units, and automated systems that assist with inventory management [57]. These applications aim to make healthcare processes in general more efficient and improve patient outcomes.

Multidisciplinary team (MDT) meetings are currently the gold standard for diagnosis of ILD [3,58]. MDTs are sometimes hybrid or completely virtual, which enables discussion of patients between community sites and ILD expert centers. Since the COVID-19 pandemic virtual MDTs are more widely implemented [59]. In Japan, a secured nationwide cloud-based integrated database has been created, in which radiological, clinical and pathological data is collected to facilitate virtual MDTs. This cloud-based database was accessible to all healthcare professionals involved. Solutions like this can improve feasibility of multicenter MDTs as data do not have to be transferred between institutions [60^▪▪^].

Another field where the IoMT is emerging, is radiology in ILD. Radiomics is the quantitative analysis of imaging data, using automatic or semi-automatic software for the purpose of extracting information from images [61]. Machine learning algorithms have been able to identify ILD in cohorts with at-risk populations [62,63]. A recent study showed the ability of an AI algorithm to detect pulmonary fibrosis on chest radiography with a sensitivity and specificity superior to that of radiologists and pulmonologists [64]. In addition, AI technology can quantify the extent of lung fibrosis, define CT-patterns, and use this information for prediction of lung function decline and mortality [65,66]. To translate this to clinical practice, large CT databases are needed, which require collaboration between institutions and across borders. External validation studies are also needed to confirm these findings in real-world clinical settings [67].

## FUTURE STEPS

Although the COVID-19 pandemic has led to an increase in use of technology in healthcare, including for patients with ILD, this has not yet led to widespread implementation of these applications, while both patient and healthcare providers acknowledge the additional value of digital care and home monitoring [28,68]. One of the main challenges for widespread implementation is the lack of reimbursement in many countries, as most funding opportunities for digital solutions during the pandemic were temporary [69]. However, there are some examples of structural reimbursement like in Germany, where some digital health applications are now reimbursed by the health insurance system [70].

Moreover, there are legislation issues that need to be addressed. The IoMT has the ability to link and interconnect healthcare professionals, and can support data sharing. Whereas data are traditionally stored on-site in hospitals, the IoMT facilitates storage of data in secured online clouds. This way it enables data sharing across regional, national, and international borders. Regulatory frameworks entailing cybersecurity, encryption protocols, and legal ownership of data, are required to expand the use of online clouds. This is especially needed for combining data from different digital platforms and for the integration of home monitoring data with in-hospital electronic patient records. In this way, large datasets, with integrated AI algorithms have the potential to optimize care processes for individual patients in the near future. Some applications of the IoMT in ILD are still in its infancy, and need further study in larger real-world cohorts to confirm and clinically validate promising results from pilot studies and translate these to daily clinical practice.

## CONCLUSION

In the last decade, the technological developments have rapidly altered the ILD landscape. We expect that in the next 10 years, AI and other innovative technologies facilitated by the IoMT will further enhance individually targeted treatment for patients with ILD by interlinking and combining data from various sources.

## Acknowledgements

*None.*

### Financial support and sponsorship

*G.N.: Financial grant from Boehringer Ingelheim paid to institution.*

*C.M.: Financial grant from Boehringer Ingelheim, Astra—Zeneca, Daiichi-Sankyo all paid to institution. Payments for presentations/lectures from Boehringer-Ingelheim and Hoffman—la Roche all paid to institution.*

*M.W.: Financial grant from Boehringer Ingelheim, Hoffman—La Roche, The Netherlands Organization for Health Research and Development, The Dutch Lung Foundation; The Dutch Pulmonary Fibrosis, all paid to institution.*

*Consulting fees from Bristol Myers Squibb, Boehringer Ingelheim, Galapagos, Galecto, Hoffman la Roche, Horizon therapeutics, Kinevant Sciences, Molecure, Nerre Therapeutics, Novartis, PureTech Health, and Respivant, all paid to institution.*

*Support for attending meeting from Boehringer Ingelheim, Hoffman la Roche, Galapagos. Participation on advisory board from Savara, Galapagos, all paid to institution.*

*Leadership role as Chair of the Idiopathic Interstitial Pneumonia group of the European Respiratory Society, Member of the board of the Netherlands Respiratory Society, Member of the scientific advisory board of the European Idiopathic Pulmonary Fibrosis and related disorders federation, Chair of the educational committee of the European Reference Network for rare Lung Diseases, Advisory board of the Dutch Lung fibrosis and Sarcoidosis patient associations.*

### Conflicts of interest

*None related to the content of this manuscript.*

## REFERENCES AND RECOMMENDED READING

Papers of particular interest, published within the annual period of review, have been highlighted as:

▪ of special interest
▪▪ of outstanding interest
